# Acupuncture improves the symptoms, gut microbiota, metabolomics, and inflammation of patients with chronic obstructive pulmonary disease: a multicenter, randomized, sham-controlled trial protocol

**DOI:** 10.3389/fmed.2025.1511275

**Published:** 2025-03-03

**Authors:** Yilin Liu, Qin Luo, Junqi Li, Chunyan Yang, Fengyuan Huang, Guixing Xu, Fanrong Liang

**Affiliations:** ^1^Acupuncture and Tuina School/The 3rd Teaching Hospital, Chengdu University of Traditional Chinese Medicine/Clinical Research Center for Acupuncture and Moxibustion in Sichuan Province, Chengdu, China; ^2^Hospital of Chengdu University of Traditional Chinese Medicine, Chengdu, Sichuan, China

**Keywords:** acupuncture, chronic obstructive pulmonary disease, gut microbiota, immune inflammatory responses, protocol

## Abstract

**Background:**

Chronic obstructive pulmonary disease (COPD) is a common chronic respiratory disease. The occurrence of COPD is associated with gut microbiota, meticulous metabolism and inflammation. Acupuncture may be effective as an adjunctive therapy for COPD, but the available evidence is limited. This study aims to confirm whether acupuncture therapy has an adjunctive therapeutic effect on COPD and to investigate the relationship between the efficacy and the gut microbiota, metabolomics and inflammation.

**Methods:**

This study is a multicenter randomized controlled trial. A total of 72 patients with stable COPD eligible will be randomized in a 1:1 ratio to receive either manual acupuncture (MA) or sham acupuncture (SA) without puncturing the skin. There will be no changes to the essential medicines used for all patients. The intervention will be 12 weeks, 3 times per week and follow-up will be 52 weeks. The primary outcome will be the change in COPD Assessment Test (CAT) score before and after treatment. Secondary outcomes will include modified Medical Research Council (mMRC), St. George’s Respiratory Questionnaire (SGRQ), 6-min walk test (6MWT), and the number of moderate or severe acute exacerbations during follow-up. A total of 36 healthy volunteers will also be recruited as normal control. In addition, feces and blood will be collected from each participant to characterize the gut microbiota, metabolomics, immune cells and inflammatory cytokines. Differences between COPD patients and healthy participants will be observed, as well as changes before and after treatment in MA and SA groups. Ultimately, the correlation among gut microbiota, metabolomics, immune cells, inflammatory cytokines and clinical efficacy in COPD patients will be analyzed.

**Discussion:**

This study will evaluate the efficacy and provide preliminary possible mechanisms of acupuncture as an adjunctive therapy in treating COPD. In addition, it will identify biomarkers of the gut microbiota, metabolites, immune cells, and inflammatory cytokines associated with therapeutic efficacy. The results of this study will be published in a peer-reviewed journal.

## 1 Introduction

Chronic obstructive pulmonary disease (COPD) is mainly characterized by airway obstruction and airflow limitation, and the main clinically manifestations include chronic cough, sputum and shortness of breath after activity, leading to a serious decline in the life quality and working ability of patients (1, 2). There are more than 300 million COPD patients in the world, and about 99.9 million in China (3). COPD has become the third leading cause of death in the world after ischemic heart disease and stroke, with more than 3 million deaths annually (4, 5). It is estimated that by 2060, the number of deaths from COPD and related diseases will exceed 5.4 million per year (6). The burden of disease caused by COPD is the fifth highest in the world, with per capita direct medical costs in China ranging from 72 to 3,565 dollars per year, accounting for 33.33 to 118.09% of annual per capita income (7, 8). The bronchodilators, inhale corticosteroids (ICS), mucolytics as well as antibiotics are currently the mainstay of COPD treatment (7), with the advantage of rapid short-term relief of dyspnea (9), but not reversal of the COPD disease process (10, 11). The improvement in clinical outcomes is based on long-term maintenance of the medication, but the patients need to bear the risk of adverse effects such as bacterial resistance (12), lung infections (13), and hearing loss (14). Furthermore, the administration of these drugs is often complicated and difficult for older and less-educated patients to acquire and adhere to (15). The financial burden of high drug prices is also difficult to ignore (16). Therefore, it is imperative to find economical and effective treatments with fewer adverse effects.

Acupuncture, as part of traditional Chinese external medicine, is widely used in the management of various diseases and considered to be one of the ideal complementary alternative therapies for COPD. Existing evidence of randomized controlled trials and systematic reviews suggests that acupuncture is effective in improving dyspnea symptoms, exercise tolerance, quality of life scores, and lung function in COPD patients compared with sham acupuncture (17). Acupuncture-assisted treatment of COPD can effectively improve life quality of patients and is more effective than applying conventional drug therapy alone (18–20). However, there are some shortcomings in the available clinical studies: for example, vague description of research methods, insufficiently reported details of the specific acupuncture/sham acupuncture method setup in the interventions, short follow-up period, and few reports on safety. Moreover, the underlying mechanism of the efficacy of acupuncture remains poorly defined.

The pathogenesis of COPD is closely associated with immune dysfunction and persistent inflammation, in which the immune homeostasis of Th17 cells and Treg cells plays an important role (21, 22). Th17 cells, named after their specific secretion of interleukin 17 (IL-17), mediate immune defense, which recruits and activates neutrophils and promotes the release of inflammatory factors (23). Treg cells mediate immune tolerance and inflammatory suppression, and negatively regulate immune cell activation and proliferation (24). Under normal body function, the two are in dynamic balance to maintain natural immune homeostasis. In pathological conditions, over-activation of Th17 cells and insufficient Treg cells can lead to hypersecretion of airway mucus and persistent inflammation, resulting in worsening of COPD (25).

The gut microbiota and the metabolome are also important in the progression of COPD (26). Studies have shown that the fecal microbiome of COPD patients is different from that of healthy individuals (27). Streptococcus, streptococcus vestibularis, and lachnospiraceae are associated with reduced lung function (28). Human commensal bacteria can ferment dietary fiber into short-chain fatty acids (SCFA), which can activate immune cells via the NLRP3 inflammatory vesicle, affecting lung inflammation and promoting COPD progression (29, 30). Metabolomics analyses have revealed that abnormalities in amino acid metabolism, lipid metabolism, energy production, as well as oxidative and antioxidant abnormalities affect the pathogenesis of COPD (31, 32). Elevated serum levels of lysine, pyruvate, 3-hydroxybutyrate, and glutamine are strongly related to acute exacerbations in patients with COPD (33). Mammalian target of rapamycin (mTOR) is a critical enzyme body that regulates cell proliferation and metabolic processes. It has been found that certain metabolites can activate the mTOR pathway and promote the differentiation of Th17 cells, as well as bind to scaffolding proteins and regulatory proteins to form mTOR complexes, induce the expression of the hypoxia-inducible factor-1α (HIF-1α), and mediate the proteasomal degradation of the forkhead-box protein P3 (FOXP3, the key transcription factor in Treg cells) to inhibit the function of Treg cells (34). Evidence suggests that acupuncture may have a therapeutic effect on COPD by decreasing the levels of tumor necrosis factors-γ (TNF-γ) and IL-4 (35), which are related to the balance of the Th17/Treg pattern. Meanwhile, acupuncture has a certain regulatory effect on gut microbiota and metabolites (36, 37). However, there are no studies linking chronic inflammation, intestinal flora, and metabolomics to investigate the mechanism of acupuncture to improve COPD from a multi-omics perspective.

Therefore, a randomized, parallel-controlled and long-follow-up trial is designed to further validate the clinical efficacy of acupuncture as an adjunctive therapy for patients with COPD and to explore the relationship between clinical efficacy and gut microbiota, metabolome and inflammation.

## 2 Materials and methods

### 2.1 Objectives

This trial aims to:

(1) Evaluate the immediate and long-term efficacy and safety of acupuncture in treating COPD;
(2) Investigate whether acupuncture can improve the clinical symptoms of COPD patients by influencing the gut microbiota and metabolome, regulating the dynamic balance of immune cells Th17/Treg and the levels of related inflammatory cytokines.

### 2.2 Trial design and setting

This is a prospective, parallel-controlled, multicenter, randomized controlled trial with blinded participants, outcome assessors, and statistician. A total of 72 COPD patients eligible will be randomly assigned to the manual acupuncture (MA) group and sham acupuncture (SA) group in a 1:1 ratio. The entire study period consists of 1 week of screening, 12 weeks of treatment and 52 weeks of follow-up. Both groups of patients will be assessed on the relevant scales throughout the study cycle (including at the time of enrollment, during the intervention period and the follow-up period), and changes in the clinical symptoms of COPD patients will be observed to clarify the effect of acupuncture on COPD. Furthermore, 36 healthy volunteers will also be recruited as a normal control group and will not receive any treatment. The trial will be conducted in four sub-centers: the Affiliated Hospital of Chengdu University of Traditional Chinese Medicine, the Sichuan Provincial People’s Hospital, the Chengdu Second People’s Hospital, and the Meishan Hospital of Traditional Chinese Medicine.

**FIGURE 1:**
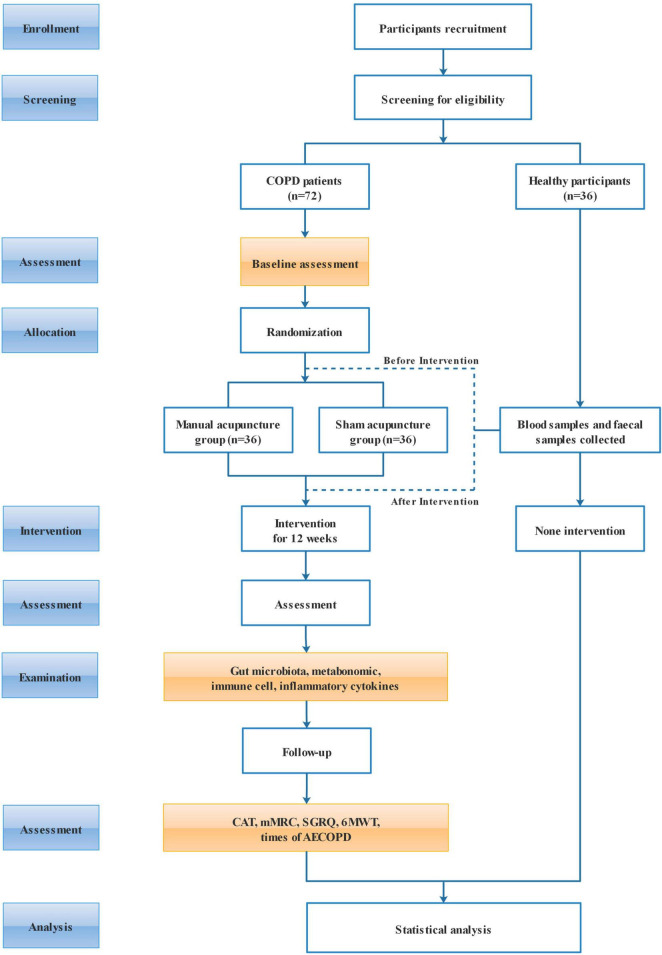
Research flow chart.

The study protocol is developed following The Consolidated Standards of Reporting Trials (CONSORT) Framework (38) and The Standards for Reporting Interventions in Clinical Trials of Acupuncture (STRICTA) (39), and will be reported in accordance with the Standard Protocol Items: Recommendations for Interventional Trials (SPIRIT) guidelines and checklist (40). The flow chart of trial procedures is shown in Figure 1, and the study schedule is depicted in Table 1.

**TABLE 1:** Schedule of enrollment, intervention, and assessments.

	Study period							
**Items**	**COPD participants**							**Healthy participants**
	**Enrollment**	**Baseline**	**Intervention phase**			**Follow-up**		**Enrollment**
	**Week -1**	**Week 0**	**Week 4**	**Week 8**	**Week 12**	**Week 38**	**Week 64**	**Week -1**
**Enrolment**								
Eligibility screen	×							×
Informed consent		×						×
Demographic characteristics		×						×
Disease history		×						×
Combined disease		×						×
Medication history		×						×
Randomization		×						
**Intervention**								
Acupuncture group			×	→	×			
Sham acupuncture group			×	→	×			
**Examinations**								
Blood detection		×			×			×
Feces detection		×			×			×
**Assessments**								
CAT		×	×	×	×	×	×	
mMRC		×	×	×	×	×	×	
SGRQ		×	×	×	×	×	×	
6-MWT		×			×	×	×	
Times of AECOPD						×	×	
Blinding assessment					×			
Safety assessment			×	×	×			
Adherence evaluation		×	×	×	×	×	×	

COPD, chronic obstructive pulmonary disease; CAT, COPD Assessment Test; mMRC, modified Medical Research Council; SGRQ, St. George’s Respiratory Questionnaire; 6MWT, 6-min walk test; AECOPD, acute exacerbation of chronic obstructive pulmonary disease.

### 2.3 Participants

#### 2.3.1 Source of participants

Recruitment strategies include posters, respiratory outpatients and previous inpatients. Patients will be informed of study specifics, including study purpose, subgroup status, interventions, treatment period, benefits, and potential risks. Patients interested in participating in this study will be critically judged and admitted by respiratory specialists based on inclusion and exclusion criteria and an informed consent form must be obtained prior to randomization into groups. All participants information will be kept confidential.

#### 2.3.2 Eligibility criteria for patients with COPD

##### 2.3.2.1 Inclusion criteria

Patients who meet the following criteria will be included (Table 2):

**TABLE 2:** Eligibility criteria.

COPD participants
**Inclusion criteria**
Patients who meet the following criteria will be included: (1) Diagnosis of COPD according to the *Global Initiative for Chronic Obstructive Lung Disease (GOLD) 2022 Report*; (2) Age between 40 and 80 years (no gender restrictions); (3) Pulmonary function tests: FEV1 between 25 and 80% after inhaled bronchodilators; (4) At least 1 moderate or severe acute exacerbation within the past year; (5) Stable condition without acute exacerbation in the last 4 weeks; (6) Being under regular medical treatment in accordance with the GOLD recommended dosing regimen within 3 months prior to enrollment, or not yet receiving any treatment; (7) Volunteering to cooperate with the study and sign an informed consent form.
**Exclusion criteria**
Patients who meet any of the following conditions will be excluded: (1) Being with severe cardiovascular, neurological, hematological, immune system, and malignant tumor diseases; (2) Being with other respiratory diseases that have a significant impact on the study (active tuberculosis, bronchial asthma, severe bronchiectasis, primary pulmonary hypertension, interstitial lung disease, etc.); (3) History of segmentectomy, wedge resection, lobectomy, total pneumonectomy, or lung volume reduction (including bronchoscopic lung volume reduction) for COPD; (4) Requiring continuous oxygen therapy according to long-term oxygen therapy (LTOT) criteria (oxygen therapy duration > 15 h/d); (5) Unable to perform pulmonary function tests, walk independently, or cooperate in completing questionnaires; (6) Presence of persistent breakage or infection of skin tissue at the site of needling, coagulation dysfunction, skin allergies; (7) Having participated in other clinical trials or performed acupuncture for respiratory disease within the past 3 months; (8) Application of antibiotics within the past 3 months; (9) Application of systemic glucocorticoids within the past 1 month; (10) Long-term application of probiotics and prebiotics; (11) Suffering from severe gastrointestinal disorders and gut microbiota disorders; (12) Being pregnant or in lactation.
**Healthy participants**
**Inclusion criteria**
Participants who meet the following criteria will be included as normal control: (1) Age between 40 and 80 years (no gender restrictions); (2) Not in sub-healthy state according to the “Clinical Guidelines of Chinese Medicine on Sub-health” of China Association of Chinese Medicine; (3) No diagnosis of respiratory system diseases, digestive system diseases, and other conditions or states of obvious clinical significance within 1 year prior to enrollment; (4) Volunteering to cooperate with the study and sign an informed consent form.
**Exclusion criteria**
Participants who meet any of the following criteria will be excluded from the normal control group: (1) Regular application of probiotics, prebiotics, or antibiotics; (2) Having gastrointestinal symptoms in the last week; (3) Being pregnant or in lactation; (4) Being in other clinical trials.

COPD, chronic obstructive pulmonary disease.

(1) Diagnosis of COPD according to the Global Strategy for the Diagnosis, Management, and Prevention of Chronic Obstructive Lung Disease: the GOLD science committee report 2022 (41);
(2) Age between 40 and 80 years (no gender restrictions);
(3) Pulmonary function tests: FEV1 between 25 and 80% after inhaled bronchodilators;
(4) Stable condition without acute exacerbation in the last 4 weeks;
(5) Being under regular medical treatment in accordance with the GOLD recommended dosing regimen within 3 months prior to enrollment, or not yet receiving any treatment;
(6) Volunteering to cooperate with the study and sign an informed consent form.

##### 2.3.2.2 Exclusion criteria

Patients who meet any of the following conditions will be excluded:

(1) Being with severe cardiovascular, neurological, hematological, immune system, and malignant tumor diseases;
(2) Being with other respiratory diseases that have a significant impact on the study (active tuberculosis, bronchial asthma, severe bronchiectasis, primary pulmonary hypertension, interstitial lung disease, etc.);
(3) History of segmentectomy, wedge resection, lobectomy, total pneumonectomy, or lung volume reduction (including bronchoscopic lung volume reduction) for COPD;
(4) Requiring continuous oxygen therapy according to long-term oxygen therapy (LTOT) criteria (oxygen therapy duration >15 h/d) (42);
(5) Unable to perform pulmonary function tests, walk independently, or cooperate in completing questionnaires;
(6) Application of antibiotics within the past 3 months;
(7) Application of systemic glucocorticoids within the past 1 month;
(8) Long-term application of probiotics and prebiotics;
(9) Suffering from severe gastrointestinal disorders and gut microbiota disorders;
(10) Presence of persistent breakage or infection of skin tissue at the site of needling, coagulation dysfunction, skin allergies;
(11) Having participated in other clinical trials or performed acupuncture for respiratory disease within the past 3 months;
(12) Being pregnant or in lactation.

#### 2.3.3 Eligibility criteria for healthy participants

##### 2.3.3.1 Inclusion criteria

Participants who meet the following criteria will be included as normal control:

(1) Age between 40 and 80 years (no gender restrictions);
(2) Not in sub-healthy state according to the “Clinical Guidelines of Chinese Medicine on Sub-health” of China Association of Chinese Medicine (43);
(3) No diagnosis of respiratory system diseases, digestive system diseases, and other conditions or states of obvious clinical significance within 1 year prior to enrollment;
(4) Volunteering to cooperate with the study and sign an informed consent form.

##### 2.3.3.2 Exclusion criteria inclusion criteria

Participants who meet any of the following criteria will be excluded from the normal control group:

(1) Regular application of probiotics, prebiotics, or antibiotics;
(2) Having gastrointestinal symptoms in the last week;
(3) Being pregnant or in lactation;
(4) Being in other clinical trials.

### 2.4 Sample size, randomization and blinding

#### 2.4.1 Sample size calculation

The primary outcome for this study is the change in the COPD Assessment Test (CAT) scores before (week 0) and after (week 12) the intervention. Sample size calculation is based on the results of our pilot trial, which showed a mean change in CAT scores of 5.0 for patients in the acupuncture group and 1.4 in the sham acupuncture group. Using a two-tailed test with type I error of 0.05 and power of 80%, a sample size of 32 cases per group is calculated with a maximum of 10% of participants allowed to drop out or disengage, for a required sample size of 36 cases per group, and a total of 72 participants will be included. Furthermore, 36 healthy participants will be included. The sample size calculation was completed in PASS 15.0 with One-Way Analysis of Variance (ANOVA) F-Tests module.

#### 2.4.2 Randomization and allocation concealment

COPD patients will be randomly divided into MA and SA groups according to a random number table generated by computer. The random sequences will be sealed in light-tight envelopes, which will be opened only when eligible participants are enrolled. Generation and allocation of the random sequences will be performed independently by a research assistant (RA) not be involved in the treatment and outcome assessment.

#### 2.4.3 Blinding

Patients, outcome assessors, data entry clerks and statistical analysts will be blinded. Letters A and B will be used instead of specific group names, and the same manipulation device will be applied to mask the difference in acupuncture between the two treatment groups of patients. During treatment, patients will be arranged to be in different rooms to avoid communication. The investigators, therapists, efficacy assessors and data statisticians will be separated throughout the whole study process. At the end of the 12-week treatment, patients will be asked if they received traditional acupuncture (yes, no, or unclear) to determine if the blinding was successful.

### 2.5 Intervention and comparison

#### 2.5.1 Patients with COPD

**FIGURE 2:**
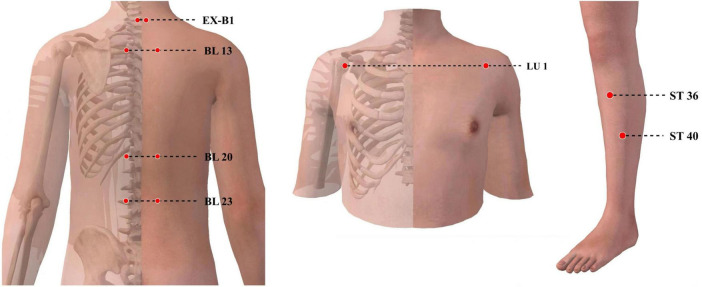
Locations of acupoints.

**FIGURE 3:**
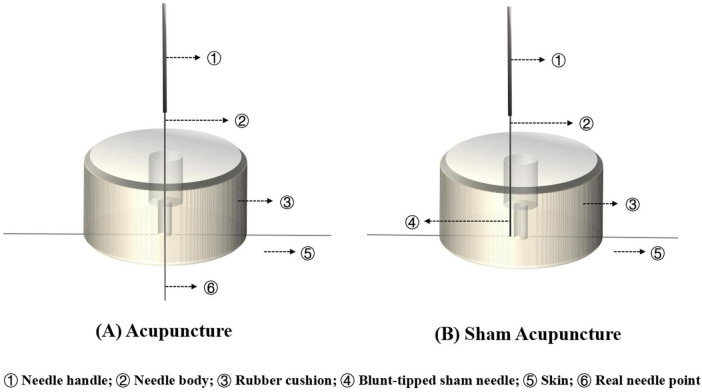
The diagram of acupuncture devices. **(A)** Acupuncture; **(B)** Sham Acupuncture. ① Needle handle; ② Needle body; ③ Rubber cushion; ④ Blunt-tipped sham needle; ⑤ skin; ⑥ Real needle point.

Details of the acupuncture intervention are reported based on the STRICTA check list in Table 3. According to the results of the existing literature collation and expert consultation, 7 pairs of acupoints are screened for treatment, including: Zhongfu (LU1), Feishu (BL13), Pishu (BL20), Shenshu (BL23), Zusanli (ST36), Fenglong (ST40), and Dingchuan (EX-B1). All acupoints are taken bilaterally, and the number of needles inserted is 7 × 2, for a total of 14 acupoints (needles). Details of each acupoint are shown in Table 4 and Figure 2. The acupuncture device same in appearance will be applicated uniformly for both MA and SA group. A rubber cushion is designed to be attached to each acupoint, and acupuncturists will operate through the cushion so that patients or bystanders cannot tell whether the needles are actually inserted or not (Figure 3). Each sub-center will be assigned an acupuncturist responsible for the treatment of all patients within that center. Acupuncturists are required to be licensed with at least 3 years of clinical experience. The expected and actual number of treatments for each patient will be recorded for assessing patient compliance.

**TABLE 3:** STRICTA checklist.

Item	Item number	Detail
1. Acupuncture rationale	(1a) Style of acupuncture	Traditional Chinese medicine
	(1b) Reasoning for treatment provided, based on historical context, literature sources, and/or consensus methods, with references where appropriate	Acupuncture treatment based on the theory of TCM, literature sources, and clinical experience in acupuncture and COPD
	(1c) Extent to which treatment was varied	Standardized acupuncture treatment
2. Details of needling	(2a) Number of needle insertions per subject per session (mean and range where relevant)	14
	(2b) Names (or location if no standard name) of points used (uni/bilateral)	Zhongfu (LU 1), Feishu (BL 13), Pishu (BL 20), Shenshu (BL 23), Zusanli (ST 36), Fenglong (ST 40), Dingchuan (EX-B1) All points will be taken bilaterally.
	(2c) Depth of insertion, based on a specified unit of measurement, or a particular tissue level	From 10 to 30 mm
	(2d) Response sought (e.g., de qi or muscle twitch response)	The “de qi” sensation will be achieved by lifting and thrusting combined with twirling and rotating the needles.
	(2e) Needle stimulation (e.g., manual, electrical)	Manual acupuncture
	(2f) Needle retention time	30 min
	(2g) Needle type (diameter, length, and manufacturer or material)	Sterile, disposable stainless acupuncture needle, 0.25 mm × 40 mm (Hwatuo, Suzhou Medical Co. Ltd., Jiangsu, China).
3. Treatment regimen	(3a) Number of treatment sessions	36 treatment sessions in acupuncture groups.
	(3b) Frequency and duration of treatment sessions	3 times a week, 30 min for each session.
4. Other components of treatment	(4a) Details of other interventions administered to the acupuncture group (e.g., moxibustion, cupping, herbs, exercises, lifestyle advice)	The standard treatment of COPD is based on the *Global Strategy for the Diagnosis, Management, and Prevention of Chronic Obstructive Lung Disease: the GOLD science committee report 2022*.
	(4b) Setting and context of treatment, including instructions to practitioners and information and explanations to patients.	The trial will be conducted in four sub-centers, all of which are the highest level hospitals in the Mainland Chinese healthcare system. All information and explanations will be provided to participants.
5. Practitioner background	(5) Description of participating acupuncturists (qualification or professional affiliation, years in acupuncture practice, other relevant experience)	All acupuncturists are qualified Chinese medicine practitioners with a degree in acupuncture and at least 3 years of clinical experience, and training in standard operating procedures for acupuncture in COPD.
6. Control or comparator interventions	(6a) Rationale for the control or comparator in the context of the research question, with sources that justify this choice	Sham acupuncture is chosen as the control group to reflect the effect of acupuncture-assisted treatment of COPD as much as possible.
	(6b) Precise description of the control or comparator. If sham acupuncture or any other type of acupuncture-like control is used, provide details on items 1 to 3 above.	The SA group will use the same acupuncture points, operating rituals and procedures as the MA group, as well as the same appearance of the needling device. However, the actual needles used are flat needles that will not pierce the skin and generate the de-qi sensation.

TCM, traditional Chinese medicine; COPD, chronic obstructive pulmonary disease; SA, sham acupuncture; MA, manual acupuncture.

**TABLE 4:** Locations of acupoints.

Acupoints	Locations
Zhongfu (LU 1)	On the upper lateral chest, 6 cun from the middle of the chest, level in the first intercostal space.
Dingchuan (EX-B1)	0.5 cun lateral to DU 14.
Feishu (BL 13)	1.5 cun lateral to the depression below the spinous process of the 3th thoracic vertebra.
Pishu (BL 20)	1.5 cun lateral to the depression below the spinous process of the 11th thoracic vertebra.
Shenshu (BL 23)	1.5 cun lateral to the depression below the spinous process of the 2th lumbar vertebra.
Zusanli (ST 36)	3 cun directly below ST 35, and one finger-breadth lateral to the anterior border of the tibia.
Fenglong (ST 40)	One finger-breadth lateral to ST 38, and at the midpoint of the line joining ST 35 and the tip of the external malleolus.

##### 2.5.1.1 Manual acupuncture (MA) group

Patients in the MA group will choose a prone treatment position. After skin disinfection, the acupuncturist will insert the needle into the acupoint through the rubber base adhered to the acupoint. The direction and angle of acupuncture will be strictly under the basic requirements of acupuncture operation. 0.5-cun acupuncture needles (0.25 × 13 mm, Hwato, Suzhou, China) will be used to insert in a depth of 10–15 mm for the BL13. 1-cun acupuncture needles (0.25 mm × 25 mm, Hwato, Suzhou, China) will be used to insert in a depth of 15–20 mm for the EX-B1, LU1, and BL20. 1.5-cun acupuncture needles (0.25 mm × 40 mm, Hwato, Suzhou, China) will be used to insert in a depth of 25–30 mm for the BL23, ST36, and ST40. After needle insertion, a modest twisting and lifting maneuver will be performed to obtain a sensation of *deqi* (soreness, numbness, distension or heaviness) (44). The degree of *deqi* sensation will be assessed immediately after the patient having *deqi* sensation (45). The needles will be retained for 30 min each time, and *deqi* sensation operation will be performed every 10 min for 15 s each time. Treatments will be given 3 times per week (1–2 days apart) for a total of 12 weeks.

##### 2.5.1.2 Sham acupuncture (SA) group

The SA group will select the same acupoints and treatment position as the MA group. The acupuncturist will applicate a special 0.5-cun flat-tipped needle (0.3 mm × 0.13 mm) to puncture vertically into the base of the rubber cushion so that the body of needle can be fixed on the patient’s skin surface. The blunt needles can make patients feel the pain without puncturing their skin. The duration of needle retention is the same as that of the MA group (30 min). A manipulation ritual of twisting and lifting like the MA group will be performed after fake insertion and every 10 min during the retention period, but without producing a *deqi* sensation, to simulate real treatment as far as possible. Equally, the intervention will be taken 3 times per week (1–2 days apart) for a total of 12 weeks. Considering the ethical requirements, patients in SA group will receive free compensatory acupuncture treatment at the end of the trial.

##### 2.5.1.3 Basic treatment and medication changes

During the baseline period, all details of medications used by the patients to treat COPD and other underlying conditions will be recorded on a case report form (CRF), including the name of the medication, the dosage used, the frequency of use, the route of administration, and when the medications begin and end. Any changes in the patient’s medications throughout the study period will also be recorded in detail. If the patient experiences an acute exacerbation, the possibility of continuing acupuncture treatment will be assessed by a respiratory medicine specialist. If an acute exacerbation occurs that necessitates the application of antibiotics or hospitalization to control the infection, the trial will be discontinued and the time and reason for being discontinued will be recorded by the investigators.

#### 2.5.2 Healthy participants

Healthy participants will receive no treatment, but only the general information registration, a corresponding health physical examination, and collection of fecal and serum.

### 2.6 Outcome measurements

#### 2.6.1 Primary outcome

COPD Assessment Test (CAT) The change in the CAT scores before (week 0) and after (week 12) the intervention is chosen as the primary outcome.

The scale evaluates and reflects the severity of COPD patients’ conditions through 8 items, including 3 indicators of clinical symptoms: cough, sputum, and chest tightness, 2 indicators of tolerance: exercise tolerance, daily activities (status of limited activities at home), and 3 indicators of life quality: sleep quality, energy, and mood (confidence in outings). The response values of each item are ranging from 0 to 5 (0 = no impairment). Response values for all 8 items will be summed up to obtain the total scores of (ranging from 0 to 40). Higher scores indicate a greater impact of the disease on the patient’s health and life (46): 0–9 is considered a low impact, 10–20 is considered a moderate impact, 21–30 is considered a high impact, and 31–40 is considered a very high impact (47). In addition, week 4, week 8 and the follow-up period (week 38, week 64) will also collect data on this scale’s scores (Table 1).

#### 2.6.2 Secondary outcomes

##### 2.6.2.1 Indicators reflecting clinical symptoms

(1) Modified Medical Research Council (mMRC): The mMRC scale will be applied to assess the dyspnea status of COPD patients. The scale is categorized into five grades (0 to 4) according to the level of activity taken when the dyspnea happens, with grade 0 (the least severe) indicating that the patient experienced dyspnea only during strenuous exercise, and grade 4 (the most severe) indicating that the patient experienced dyspnea with just the slightest activity (48).

(2) St. George’s Respiratory Questionnaire (SGRQ): The SGRQ will be applied to assess the life quality of COPD patients. The scale consists of two parts, reflecting the impact of COPD on patients from three dimensions: the first part contains symptomatic questions, answered in the form of single-choice questions, which ask patients about the frequency and severity of the occurrence of cough, sputum, shortness of breath, and dyspnea; the second part is based on right and wrong questions, which include questions about activities (activity limitation due to dyspnea) and the impact of the disease (impaired socialization and psychological disorders due to airway disease). The 3 main dimensions is scored according to the weighting of the different questions, with the scores calculated using a weighted average method, resulting in a symptom score, an activity score, and an impact score, which are finally summarized to give a total score. Higher patient scores indicate poorer quality of life (49).

(3) 6-min walk test (6MWT): The 6MWT will be applied to assess the exercise tolerance of COPD patients. The test is commonly used to objectively assess patients with moderate-to-severe pulmonary disease. In the test, patients will be asked to walk as far as they can for 6 min along a 30-meter-long corridor. The 6-min walk distance (6MWD) (50), heart rate, respiratory rate, and the values of oxygen saturation (SpO_2_) will be recorded before and after the test (51). The degree of dyspnea will be assessed before and after the test by the Borg scale. The Borg scale consists of a magnitude of 0–10, with 0 being “no dyspnea at all” and 10 being the “the worst dyspnea.” In conjunction with 6MWD recordings, changes in dyspnea levels at rest and after sub-extreme exercise can be assessed, as well as the patient’s exercise tolerance under daily physical activity.

(4) Number of moderate or severe acute exacerbations of during the follow-up period: The number of moderate or severe acute exacerbations in the follow-up period will be counted, as one of the indicators for assessing the long-term effects of acupuncture-assisted treatment for COPD.

The moderate (patient needs to be treated with short acting bronchodilators plus antibiotics and/or oral corticosteroids) and severe (patient requires hospitalization or visits the emergency room) acute exacerbations of COPD are defined and classified referring to the *Global Strategy for the Diagnosis, Management, and Prevention of Chronic Obstructive Lung Disease: the GOLD science committee report 2022* (41).

The measurement nodes for the outcome indicators above are detailed in Table 1.

##### 2.6.2.2 Other indicators sent for testing

Fecal samples and fasting blood samples will be collected at baseline (week 0) and at the end of the intervention (week 12) for COPD patients, and only at baseline for healthy subjects.

(1) Changes in gut microbiota: The third generation of full-length 16S rRNA sequencing technology will be utilized to analyze the gut microbiota of participants.

Participants will be advised to maintain a normal diet (no specific changes from previous habits) and to avoid spicy, greasy or probiotic-containing foods such as yogurt prior to the collection of fecal samples. The sample should be collected approximately 4 g from the middle internal part of the feces using a disposable fecal kit and stored in a −80°C refrigerator within 30 min. The DNA of gut microbiota from fecal samples will be extracted by using dedicated kits, then a SMRT (single-molecule real-time) bell library will be constructed. SMRT sequencing will be performed on the PacBio Sequel II sequencing platform (Pacific Biosciences, USA). After raw data processing, clustering or noise reduction analyses will be performed to obtain ASVs (Amplicon Sequence Variants). Based on these sequences, species annotation, further cluster analysis and difference analysis can be performed. Indicators for the analysis of gut microbiota include structural diversity of the microbiota, and expression abundance of key strains as well as their intergroup differences.

(2) Changes in content of metabolomics: The untargeted metabolomics study will be performed on blood samples from participants by using liquid chromatography-tandem mass spectrometry (LC-MS/MS) technology (52, 53).

Disposable anticoagulation tubes will be utilized to collect the peripheral venous blood samples. A portion of the blood samples will be separated by centrifugation with serum dispensed into dry tubes. Multiple tubes of each sample will be prepared for backup and stored in a refrigerator at −80°C. The serum samples will be processed accordingly to extract the metabolites and then sent to the instrument for analysis. The raw files (.raw) obtained by mass spectrometry will be imported into Compound Discoverer 3.3 software for spectrum processing and database searching to get qualitative and quantitative results of metabolites. By multivariate statistical analysis, the differences in metabolic patterns among different groups will be revealed and the screening of differential metabolites will be performed. Hierarchical clustering and metabolite correlation analyses will be used to reveal the relationships between samples and between metabolites. Finally, systematic pathway annotation of all identified metabolites and differential analysis and KEGG (Kyoto Encyclopedia of Genes and Genomes) analysis of all differential metabolites will be performed to explain the biological significance of metabolite correlations.

(3) Changes in percentage of Th17/Treg immune cells: Flow cytometry will be utilized to quantify Th17 and Treg cells from the blood samples and the ratio of the two will be calculated.

A portion of the whole blood samples will be taken to make a single cell suspension. Blood cells will be stabilized using anticoagulants and then stained for cell surface markers using flow cytometry. The labeled cells will be detected and analyzed using a fluorescein analyzer. By setting up different fluorescence channels, the proportions of Th17 and Treg cells will be detected and calculated, respectively.

(4) Changes in expression levels of inflammatory cytokines: The enzyme-linked immunosorbent assay (ELISA) will be utilized to measure the levels of the following indicators in serum samples from the participants: TNF-α, IL-17A, IL-1β, IL-2, IL-10, IL-4, IL-6, interferon-γ (IFN-γ), α7 acetylcholine (ACHα7).

Corresponding ELISA kits will be prepared. Standards and quality control solution will be prepared according to the manufacturer’s instructions. Then The samples and standards will be added separately to the ELISA plates with antibodies for the detection of specific inflammatory factors. After the steps of washing plates, adding substrate and terminating the reaction, the absorbance values will be measured based on color intensity. Finally, a standard curve will be fitted and the concentration of the inflammatory factor in the sample will be calculated in terms of the standard curve.

### 2.7 Safety assessment

Safety will be assessed by vital signs examination and routine blood tests before and after the treatment period. Adverse events (AEs) associated with the intervention, including bleeding, hematoma, needle breakage, local infection, persistent pain, syncope, etc., must be reported promptly and recorded in the CRFs, which included symptoms, signs, time of occurrence, extent, duration, disposal measures, process and results of AEs (Note: acute exacerbation of COPD is not counted as an adverse event). Serious cases should to be reported to the Project Management Office and Ethics Committee of Chengdu University of Traditional Chinese Medicine within 24 h and their participation in trial will be aborted. Upon occurrence of an adverse event, participants will immediately receive appropriate treatment and decide whether to withdraw from the trial at their own discretion. Participants who abort the trial will continue to be investigated and followed up, and the results will be recorded accordingly.

### 2.8. Management of acupuncture-related adverse events

The acupuncturist will take immediate action for adverse events related to acupuncture. In the event of minor adverse events such as localized bleeding and mild pain, the acupuncturist will apply pressure to the bleeding area to stop the bleeding and observe and reassure the painful area. In the event of a serious adverse event such as needle breakage or fainting, the patient will be quickly transported to the emergency room. Depending on the circumstances, the emergency room medical staff will take appropriate measures such as surgery, anti-infection treatment and emergency resuscitation to deal with the situation. Close monitoring of the patient’s condition and follow-up examinations will be conducted to ensure recovery.

### 2.9 Trial quality control, data management, and monitoring

Prior to the formal conduct of the trial, all study personnel will be issued with a workbook and receive standardized training to familiarize them with the implementation details of this study and to ensure consistency in the study execution process and the assessment of efficacy. They will learn uniformly how to interpret the eligibility criteria and complete the CRFs normatively. Acupuncturists will receive normative training in acupoint positioning and acupuncture operating procedures for COPD to ensure the safety of operation and effective blinding. RAs will exist and be trained independently to ensure complete randomization and allocation concealment. Clinical monitors will be set up to periodically verify that each investigator is strictly following the study protocol during the trial, and to periodically check the informed consent, inclusion and treatment progress of participants, as well as the completion of CRFs at each sub-center.

Raw clinical data will be collected by CRFs in paper version, entered and stored in a password-protected electronic database by specially trained data-entry personnel with unknown subgroups. Access to the database will be limited to specific researchers only. All paper copies of study documents will be stored centrally and locked. The Ethics Committee of the Affiliated Hospital of Chengdu University of Traditional Chinese Medicine may inspect the study records and monitor the trial.

### 2.10 Statistical analysis

Analyses of effectiveness, indicators of DNA library of gut microbiota, content of metabolites, percentage of immune cell and expression levels of inflammatory cytokines will be performed according to the Per Protocol Set (PPS) principle. The missing data will be filled in by multiple interpolation. SPSS 27.0 software (IBM Corp, New York) will be applied for statistical analysis. Quantitative indicators will be described by mean value (± standard deviation) or median (± interquartile range), and classification indicators by frequency and percentage. Baseline data will be analyzed using Chi-square or Fisher precision tests (data with frequency less than 5) and ANOVA. Sensitivity analyses will be performed using ANOVA.

The outcome of effectiveness, indicators of DNA library of gut microbiota, content of metabolites, percentage of immune cell and expression levels of inflammatory cytokines will be analyzed by linear mixed effects model. Fixed effects will be set for analyzing groups, COPD subtypes and smoking index (calculation method: smoking years × daily smoking number ÷20); Random effects for sub-centers and baseline outcome measures, both including intercept; Model-based covariance will be applied to test the fixed effects and coefficients, the degree of freedom will use residual method, and the maximum number of iterations will be set to 100. To control for type I error rates under multiple comparisons, the Benjamini–Hochberg method will be utilized for multiple comparison correction. Multiple linear regression will be used to analyze the relationship between clinical efficacy and gut microbiota, metabolites, immune cells and inflammatory factors. Statistical tests will be bilateral with *P* < 0.05 considered statistically significant.

## 3 Discussion

COPD is a major public health problem. It not only seriously affects the health and life quality of patients, but also imposes a heavy burden on families and society. There are concerns about side effects and patient compliance with the long-term use of conventional drug regimens. Therefore, the combined use of complementary alternative therapies is expected to help alleviate these problems. However, the available evidence suffers from study design flaws and the mechanisms by which acupuncture works have not been well understood. The aim of this trial is to reassess the efficacy of acupuncture as an adjuvant therapy for COPD and explore the mechanisms of acupuncture efficacy from a perspective by gut microbiota, metabolome and inflammation.

In this study, patients with stable COPD are selected as the research population. COPD has a long disease duration, with high rates of disability and death (54). And the frequent recurrence of acute exacerbations of COPD is an important factor which affects the prognosis of condition and leads to death (55). Therefore, interventions during the stabilization period to prevent and reduce acute exacerbations can result in the most meaningful management of the condition for patients.

A set of commonly used, safe and effective acupoints in the clinic are selected for this trial (56, 57). LU1 and BL13 are the back-shu point and front-mu point of the lungs, respectively, which are key points for regulating the essence and function of the lungs, as well as projections of the anatomical location of the lungs on the body surface (58). EX-B1 is a particular acupoint for the treatment of asthma and dyspnea selected based on the long-term experience of clinicians. BL20 and BL23 tonify the spleen and kidney, respectively. ST36 is the most used acupoint for treating COPD besides BL13 (57). Together with BL20 and BL23, it increases the synergistic effect of regulating the lungs, spleen and kidney. Additionally, ST36 can nourish the healthy *qi* then enhance the immunity of COPD patients during the stable period. Studies have shown that ST36 can regulate the gut microbiota and reduce inflammation in the body (59, 60). ST40 works to resolve phlegm and achieve symptomatic treatment.

The intervention period is up to 12 weeks long, with a view to maximizing the effect of acupuncture. However, in clinical practice, long-term treatment is not readily achievable given the cost of treatment and patient commuting. Therefore, the efficacy data will be collected every 4 weeks during the intervention period to explore the optimal duration of the intervention. This may provide a basis for developing a normative acupuncture protocol for the stable phase of COPD. The follow-up period of up to 1 year is designed to assess the long-term effects of acupuncture by observing the continued improvement in the clinical indicators and the number of moderate-severe acute exacerbations during the 1-year period after the end of treatment. During the study, patients will refrain from receiving any traditional Chinese medicine other than basic medication and any additional use of western medicine will be recorded in detail to minimize bias and evaluate the effect of acupuncture-assisted treatment critically.

A sham acupuncture of “real acupoints but fake insertion” is set as control for this study. The acupoints, appearance of the acupuncture device, and mode of operation will be same as in the treatment group. Thus, the necessary therapeutic experience can be provided for participants, with the actual acupuncture stimulus being controlled so as not to produce additional physiological effects. Compared to the choice of non-acupuncture points or shallow puncture, this can achieve psychological comfort and physiological inertia of the participant to a greater extent to ensure blind implementation (61).

The change in CAT scores before and after treatment is chosen as the primary outcome to comprehensively evaluating the efficacy of acupuncture as an adjuvant therapy for COPD patients from aspects of clinical symptoms, tolerability and quality of life. Although the test of pulmonary function is considered to be the gold standard for the diagnosis of COPD and an objective indicator reflecting changes in lung function (62, 63), it does not fully reflect the degree of dyspnea and severity of disease in COPD patients. There are a certain number of COPD patients having clinical manifestations without typical lung function changes (64). The CAT score is a comprehensive evaluation method, which has a relatively strong correlation with commonly used pulmonary function indicators such as FEV1 and FVC, and has high sensitivity in assessing the degree of lung damage and the incidence of complications in patients (65).

Studies have shown that gut microbiota and metabolome dysregulation are strongly associated with the development and progression of COPD (66–68). Dysregulation of the gut microbiota leads to the production of a toxic substance lipopolysaccharide (LPS) (69). LPS can cross the intestinal barrier into the body circulation and reach the lungs, where it exacerbates COPD lung inflammation through the toll-like receptor 4/ nuclear transcription factor-κB (TLR4/ NF-κB) signaling pathway mediating the production of IL-1β, IL-6, IL-17 TNF-α (70). The transplantation of gut microbiota from healthy individuals can alleviate COPD-associated systemic inflammatory responses and lung histopathological damage (71). Pulmonary inflammatory mediators of COPD patients can cause elevated CRP (C-reactive protein) and keratinocyte-derived chemokines in peripheral serum, which subsequently disrupt intestinal immunity and homeostasis (72). Altered metabolism of phospholipid-derived sphingolipids and glycerophospholipids in COPD patients can lead to accumulation of ceramide in lung tissue, followed by dysregulation of endothelial defense mechanisms, induction of apoptosis in alveolar epithelial cells, promotion of inflammatory responses and macrophage dysfunction (73). Also, increased oxidative stress, phospholipid oxidation and activation of the innate immune system will contribute to the persistence of inflammation (74). Previous studies have investigated the modulation of the gut-brain axis by acupuncture using microbiomics and metabolomics approaches, providing meaningful evidence for the treatment of a wide range of disorders including stroke, insomnia, and Alzheimer’s disease (75, 76). Electroacupuncture has been proved to increase the levels of SCFA, a metabolite of gut microbiota in an ischemic stroke rat model, and attenuate brain and gut inflammatory injury in stroke rats by enhancing SCFA-mediated Foxp3 acetylation in Treg cells (77). However, there is a lack of studies in which acupuncture modulates the immune homeostasis of gut microbiota and lungs. The study will determine the pathological characteristics of the patients in terms of gut microbiota, metabolism and immune inflammation by comparing them with healthy volunteers and observe the modulating effect of acupuncture on these and explore their relationship with the efficacy of COPD.

Some inevitable limitations exist in this study. Firstly, only patients with moderate or severe COPD will be included in this study, and the applicability of the findings to mild and very severe patients will be questionable. Secondly, many other lung diseases will be excluded, and the participants may be somewhat different from the real COPD patient population. Finally, due to the nature of the acupuncture operation, it is not possible to blind the acupuncturist. To minimize any resulting bias, this study will strictly enforce allocation concealment, and blinding of patients, assessors, and statistical analysts. Registration and measurement of outcomes will be completed by personnel unaware of the subgroups and interventions.

In conclusion, the results of this trial will hopefully provide valid clinical evidence for supporting the efficacy of acupuncture-assisted treatment for stable COPD, and on this basis, explore the relationship between the acupuncture effect and gut microbiota, metabolome, and inflammation, which will provide a scientific basis for elucidating the mechanism of action of acupuncture therapy.
